# The Effect of “Creating Opportunities for Parent Empowerment” Program on Parents of Children With Epilepsy and Other Chronic Neurological Conditions

**Published:** 2020

**Authors:** Maryam JAHRI SHEIJANI, Minoo Mitra CHEHRZAD, Shadman REZA MASOULEH, Ehsan Kazem NEZHAD LEYLI, Elham BIDABADI

**Affiliations:** 1 School of Nursing and Midwifery, Guilan University of Medical Sciences, Rasht, Iran; 2 Department of Nursing, Social Determinants of Health Research Center (SDHRC), School of Nursing and Midwifery, Instructor, Guilan University of Medical Sciences, Rasht, Iran; 3 Social Determinants of Health Research Center (SDHRC), Bio-Statistics, Guilan University of Medical Sciences, Rasht, Iran.; 4 Department of Pediatrics, Medical School, Guilan University of Medical Science, Rasht, Iran., Rasht, Iran.

**Keywords:** Epilepsy, Child, Anxiety, Parents, Creating Opportunities for Parents Empowerment

## Abstract

**Objectives:**

Parents taking care of children with epilepsy experience stress in their daily lives, which enhances their anxiety, changes their function, and eventually increases their children's behavioral problems. The present study aimed at investigating the effect of creating opportunities for parent empowerment (COPE) program on parents of children with epilepsy or other chronic neurological conditions.

**Material & Methods:**

A quasi-experimental clinical trial was conducted on 88 mothers of hospitalized children with epilepsy aged 3 to 12 years. In the intervention group, the COPE program was conducted by the researcher in three phases and the usual care *group* received *usual healthcare*. The mothers’ anxiety was assessed in three phases in both groups.

**Results:**

Results of the present study showed that the effect of time and group-time interaction on the state anxiety and trait anxiety was significant in the intervention group; however, the effect of time was not significant in the usual care group (*P* = 0.12). The differences of state anxiety and trait anxiety were not significant between the two groups (*P* = 0.136), which depended on the baseline level of anxiety. By analyzing the covariance after controlling the two variables at baseline, it was observed that the score variations in the state anxiety and trait anxiety were significant at all studied time points.

**Conclusions:**

The results of the current study indicated the positive effect of the COPE program on the anxiety of parents of children with epilepsy. Since this technique is non-pharmacological, convenient, easy-to-apply, and cost-effective, it can be used to reduce the parents’ anxiety.

## Introduction

Epilepsy is a neurological disorder characterized by persistent talent for seizures, and cognitive, psychological, and social outcomes. This disease is considered as a life-threatening condition and medical emergency followed by a high rate of mortality and illness (1). About one quarter of epilepsy cases begin in childhood (2), and as the most common chronic childhood disorder, it affects about 1% of children. A declining epilepsy prevalence is reported in high-income countries (3).

Studies show that epilepsy and other chronic neurological disorders with epileptic seizures affect physical, cognitive, social, emotional, and behavioral functions in the affected children (4). The hospital experience has the potential to have long-lasting and negative impact on the child(5, 6). The consequences of epilepsy are not limited to the affected children as they influence all family members, especially the parents as their main caregivers (7). Despite the many studies conducted on patients with epilepsy, the psychiatric impact of epilepsy on the parents of affected children are a topic not dealt with much (8). Management of a child with epilepsy is very difficult and, despite medication, some of the epileptic seizures are uncontrollable. On the other hand, children with epilepsy and other neurological disorders cannot control attacks, and this is often the responsibility of parents and, especially, the mothers (9, 10). Children with chronic neurological disorders live in conditions that are often accompanied with no prognosis. This uncertainty causes stress that reduces parents` belief in their skills and causes anxiety, depression, and eventually a function change, and thereby increases the child's behavioral problems(11).

Parents of children with chronic diseases, including epilepsy, have low quality of life. On the other hand, such children are dependent on their parents for care and health control; thus, the quality of care they receive is influenced by the parents’ well-being and feelings. As a result, the health care providers, especially nurses, should be concerned about the well-being and mental condition of the parents of children with epilepsy(12). The focus of studies is on providing medical knowledge to parents, but the limited psychosocial support offered to families as a nursing intervention is not investigated. This is an area with significant need for further research. For a successful parental intervention, it needs to address psychosocial implications, since it is known that the parent’s mental health can have a significant impact on the child’s quality of life (13). 

The "creating opportunities for parent empowerment" (COPE) program, first developed in 2001 by Bernadet melnyk, is an educational-behavioral intervention relied on self-regulation theory and control (14). The program focuses on increasing parents' knowledge and understanding of child behavioral changes during hospitalization and after being discharged from hospital as well as parents` direct involvement in the physical and emotional care of their children (15). Parents participating in the COPE intervention reported decreased levels of stress and anxiety, fewer depressive symptoms, and increased confidence in their parenting abilities compared with their counterparts that did not participate in the intervention (16-18). Families of children with epilepsy need to learn how to adapt to stress induced by uncertain prognosis, frequent hospitalization, and therapeutic procedures and visits. Parents' role in facilitating their child’s adaptation does not end with hospitalization and trainings on how to facilitate adaptation needs to be constantly empowered when a child is hospitalized or required to undergo treatment procedures. The COPE program can be useful for children with chronic diseases since it teaches parents how to diagnose and interpret their child's behaviors. The COPE’s supportive strategies facilitate the transition from hospital to home in children with chronic diseases. Transition from hospital to home is stressful for parents, since there is uncertainty about child care, and parents do not know how to help their children dealing with their experiences in the hospital. Since children with chronic diseases experience frequent hospitalization throughout their illness period, there is an information gap in this regard (19).

According to the best of authors` knowledge, no study is conducted in Iran on the effect of the COPE program on the parents of children with epilepsy, and given that parents of such children need more help and training than the parents of children with other chronic diseases and that one of the evident nursing duties in this group is training, and with regard to the low cost, safety, and efficacy of the COPE program, the current study aimed at investigating the effect of the COPE program on the anxiety level of parents of children with epilepsy and other chronic neurological disorders in order to reduce the parents' anxiety levels, and ultimately decrease the behavioral problems in such children.

## Material & Methods

Participation in the study was voluntary and the participants received complete information regarding the objectives of the study and their written informed consent was also obtained.

The current semi-experimental clinical trial was conducted on 88 mothers of 3-12-year-old children with epilepsy or other chronic neurological disorders associated with epileptic seizure referring to the education and treatment center for children in Rasht, Iran. Continuous and convenience sampling was employed and the selected subjects were randomly divided into the experimental and usual care groups. The inclusion criteria were: mothers of 3-12-year-old children with epilepsy or other chronic neurological disorders with epileptic seizures, unemployment of mothers in health system, lack of special events affecting the mothers’ level of anxiety (e g, death of a close relative or a financial crisis), mothers who are in charge of taking daily care of their children, and access to the phone. The exclusion criteria consisted of the parents' reluctance to participate in the program at any of the phases of the study. Continuous sampling was performed from December 2017 to May 2018.

The intervention group received the COPE program in three phases (Table 1). The first phase of the program was implemented when the children were hospitalized and included presentation of audio and written information on the children's expected responses to the disease and on how the cares can facilitate the children’s adaptation to the hospitalization experiences. The second phase of the intervention was performed three days after discharge from the hospital in which the researcher called the mothers in order to strengthen the information provided at the first phase of the program. The information was developed to make parents anticipate and appropriately respond to child behavioral changes when they went back home. The third phase of the intervention was implemented four weeks after discharge from the hospital in which a parent-child workbook was submitted to train mothers how to help their children adapt to the medical procedures and hospitalization using therapeutic play techniques. The usual care group, on the other hand, only received the usual healthcare services of the hospital.

**Table 1:** Intervention Timeline

Intervention Timeline		
	**Intervention Group**	**Usual Care Group**
During admission	*Signed informed consent *Completed questionnaires *Received information about the hospital unit and information on phase I of the COPE	*Signed informed consent *Completed questionnaires *Received information about the hospital unit
Three days after discharge	Received telephone call to review information given in the hospital during phase I of the COPE	Received telephone call to review satisfaction with hospital stay and discharge
One week after discharge	Completed questionnaires at home	Completed questionnaires at home
Four weeks after discharge	Received phase III information at home	-
Eight weeks after discharge	Completed questionnaires at home	Completed questionnaires at home

Regarding the objectives of the study, the data collection instruments were a demographic questionnaire and the Spielberger state-trait anxiety inventory (STAI-X). The demographic questionnaire for the children and their parents contained personal features and clinical information. The STAI-X was used to measure the mothers’ level of anxiety. The first version of the inventory was presented by Spielberger et al., and validated in Iran by Mahram(20). The STAI-X consists of two self-assessment scales with 40 items (the first 20 items on state anxiety and the second 20 items on trait anxiety). The total score ranges from 20 to 80, with higher scores representing a higher level of anxiety. Data were analyzed by SPSS version 21 using descriptive and inferential statistics such as Chi-squared, independent samples t-, the Mann-Whitney, and the Wilcoxon tests, as well as MANCOVA.

## Results

Of the 88 mothers participating in the present study, 44 were allocated into the usual care group and 44 into the intervention group. From the usual care group, 36 mothers completed the one-week-after-discharge questionnaire and 32 subjects completed the eight-week-after-discharge questionnaire; however, 37 mothers in the intervention group completed the one-week-after-discharge questionnaire and 34 mothers completed the program.

Findings of the study indicated that the majority of children with epilepsy were male (54.5%) with a mean age of 5.5 years. According to what the mothers reported, 75% of the children were able to take care of themselves and their first hospitalization was about three years ago in average. The mothers’ mean age was 32 years and about half of them had elementary and secondary school education (46.6%). Regarding the level of education, the majority of fathers had a similar status (46.6%). In the current study, the mothers were mostly housewives (86.4%) and the fathers had different occupations (60.2%) The mothers’ level of satisfaction was high with their husbands’ support (58.0%) and family support (58.0%), and many of them reported other problems in life as low (37.5%).

Based on the statistical tests, no significance difference existed between the children and parents’ demographic status in the two groups (*P *>0.05). The only statistically significant differences between the two groups based on the Fisher exact test were fathers’ levels of education (*P* = 0.033) and fathers’ occupation (*P* = 0.001).

According to Table 2, there was a significant difference in the anxiety level between the intervention and the usual care groups in one (*P* = 0.034) and eight weeks after discharge (*P* = 0.002). Such significant differences were also observed for the state anxiety between the baseline and one week after discharge (*P* = 0.015), the baseline and eight weeks after discharge (*P* <0.001), as well as one and eight weeks after discharge (P = 0.014); therefore, the anxiety level in the intervention group was higher at all studied time points than those of the usual care group. In general, according to the repeated measures ANOVA, there was a statistically significant difference in the state anxiety scores among all studied time points. The difference in the state anxiety score between the two groups was not significant in general (*P* = 0.09). This depends on the baseline level of anxiety. This co-variate was controlled to further examine the efficacy of the COPE program using the ANCOVA.

**Table 2:** Comparison of Mean ±SD Scores of the State Anxiety Inventory by Group and Time

**Group**	**Data Intervention Group** **(Mean ±SD)**	**Data Usual Care Group (Mean ±SD)**	**P-value**	**Statistical Test **
**Data**				
State (pretest)	56.52 ± 13.21	56.14 ± 10.44	0.664	The Mann-Whitney
State (one week)	44.95 ± 13.40	51.46 ± 11.39	0.034	The Mann-Whitney
State (8 weeks)	41.85 ± 12.01	51.45 ± 13.02	0.002	The Mann-Whitney
Difference between pretest versus 1-week posttest	11.27 ± 11.84	4.94 ± 9.25	0.015	The Mann-Whitney
Difference between pretest versus 8-week posttest	15.21 ± 10.63	4.58 ± 10.84	0.001	The Mann-Whitney
Difference between 1-week versus 8-week posttest	4.35 ± 6.80	0.01 ± 6.28	0.014	The Mann-Whitney
P. value, effect of time	0.001	0.11		Repeated measures ANOVA
P. value, effect of group	0.09			Repeated measures ANOVA
P-value, interaction between time and group	0.001			Repeated measures ANOVA

According to Table 3, the trait anxiety scores were significantly different in the two groups one (*P* = 0.039) and eight weeks after discharge (*P* = 0.009). Furthermore, there was a significant difference in the trait anxiety score between baseline and one week after discharge (*P* = 0.01) as well as baseline and eight weeks after discharge (*P* = 0.02) in the two groups. The only significant difference was observed for the trait anxiety score in the intervention group between the baseline (*P* = 0.679) and one to eight weeks after discharge (*P* = 0.628). Based on the repeated measures ANOVA, the time effect was (*P* <.001) in the intervention group and group-time interaction was statistically significant (*P* < 0.001); however, the time effect in the control group was not significant (*P* = 0.12). Similar to the state anxiety, the level of trait anxiety was not significantly different between the two groups (*P* = 0.136).

The results of MANCOVA and the Wilks 'Lambda test indicated that the effect of covariates (i e, state anxiety and trait anxiety scores before intervention) on the results of the state anxiety and trait anxiety was statistically significant (*P* <0.001).

The results of covariance analysis are shown based on the Bonferroni test in the following tables after controlling two variables of “state anxiety” and “trait anxiety” in the control and intervention groups.

**Table 3:** Comparison of Mean ±SD Scores of Trait Anxiety Inventory by Group and Time

**Group **	**Data Intervention Group** **(Mean ±SD)**	**Data Usual Care Group (Mean ±SD)**	**P-value**	**Statistical Test **
**Data**				
Trait (pretest)	52.82 ± 13.62	53.75 ± 11.62	0.679	Mann-Whitney
Trait (1 week)	46.27 ± 13.69	52.72 ± 11.78	0.039	Mann-Whitney
Trait (8 week)	43.79 ± 12.89	51.06 ± 11.20	0.009	Mann-Whitney
Difference between pretest versus 1-week posttest	6.00 ± 6.92	1.33 ± 7.92	0.011	Mann-Whitney
Difference between pretest versus 8-week posttest	6.00 ± 9.71	2.45 ± 7.04	0.02	Mann-Whitney
Difference between 1-week posttest versus 8-week posttest	4.00 ± 3.47	1.67 ± 5.26	0.628	Mann-Whitney
P–value, effect of time	0.001	0.12		Repeated measure ANOVA
P.-value, effect of group	0.136			Repeated measure ANOVA
P–value, interaction between time and group	0.001			Repeated measure ANOVA

According to Table 4, there was a significant difference in the state anxiety (*P* = 0.033) and trait anxiety (*P* = 0.018) scores between baseline and one week after discharge as well as the state anxiety (*P* = 0.001) and trait anxiety (*P* = 0.004) scores between baseline and eight weeks after discharge in the intervention group, compared to the control group. In addition, the difference in state anxiety score between one and eight weeks after discharge (*P* = 0.03) was significant in the intervention group compared to the control group, while no significant difference was observed in the trait anxiety score between one and eight weeks after discharge (P = 0.38) in the intervention group in comparison with the control group.

**Figure 1:**
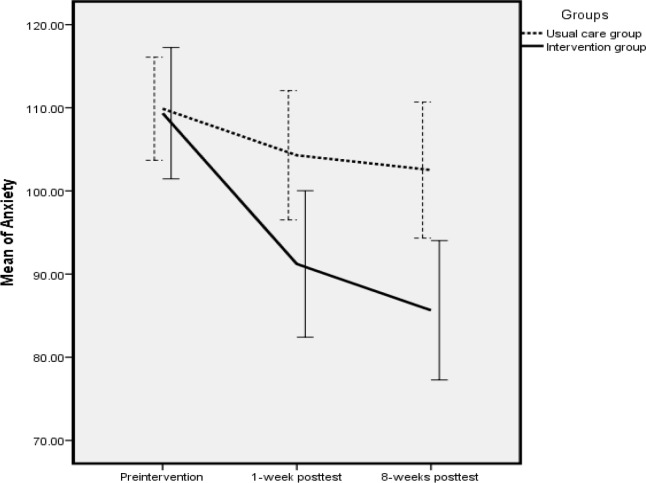
Comparison of Mean Anxiety by Group

Regarding the repeated measures ANOVA, there was a significant difference in the state anxiety score among all studied time points. This means that the variation of the anxiety scores in the intervention group was greater than those of the usual care group (Figure 1).

**Table 4:** Comparison of Difference State-Trait Anxiety Inventory Scores by Group and Time

**D** **ependent Variable**	**Mean Difference**	**Std. Error**	**95% Confidence Intermission**		**F**	**df**	**P-value**	**Observed Power**	**Effect**	**Statistical Test Used**
			**Lower Bound**	**Upper Bound**						
Difference in state anxiety scores between pretest versus 1-week posttest	4.629	2.130	0.376	8.883	4.7	1	0.033	0.572	0.067	MANCOVA
Difference in state anxiety scores between pretestversus 8-week posttest	7.538	1.979	3.578	11.494	14.49	1	0.001	0.963	0.192	MANCOVA
Difference in state anxiety scores between 1-week posttest versus 8-week posttest	3.670	1.648	0.373	6.967	4.9	1	0.03	0.591	0.076	MANCOVA
Difference in trait anxiety scores between pretest versus 1-week posttest	4.664	1.921	0.829	8.500	5.89	1	0.018	0.667	0.082	MANCOVA
Difference in trait anxiety scores between pretest versus 8-week posttest	5.822	1.921	1.981	9.664	9.18	1	0.004	0.847	0.131	MANCOVA
Difference in trait anxiety scores between 1-week posttest versus 8-week posttest	1.568	1.771	-1.975	5.111	0.784	1	0.380	0.140	0.013	MANCOVA

## Discussion

The current study findings revealed that the mothers had the highest anxiety level during the hospitalization period. Although this finding was not expected, other studies confirmed it (21). Frank et al., found that more than a quarter of parents whose children are hospitalized experience high levels of anxiety associated with the parents' negative adaptation strategies (22). Charena et al., also concluded that the parents’ high level of anxiety when their child was hospitalized was correlated with their lower levels of education (23). These findings were not unexpected since the literature supported that the time of diagnosis and hospitalization is the time of greatest stress for parents (24, 25). Parenting a child acutely hospitalized with a neurological diagnosis produces anxiety related to management of the child’s condition. This anxiety is often attributed to the uncertainty of a child’s prognosis when they are admitted to the hospital to manage his/her neurological symptoms (26).

Additionally, the findings of the present study suggested that the implementation of the COPE program reduces anxiety in mothers of children with epilepsy and a statistically significant difference existed between the two experimental and usual care groups in terms of reduced level of anxiety before the intervention, and one week and eight weeks after discharge from hospital. With regard to the effect of the COPE program on the anxiety level of parents of children with epilepsy and other chronic neurological diseases in the current study, the findings were in line with those of Duffy et al. (2016). In their study, they aimed to examine the impact of the COPE program on parents in Boston, USA, and claimed that the COPE intervention reduced parents’ anxiety level in the intervention group as the differences between the intervention and control groups were statistically significant (*P* = .005). The mean score of anxiety decreased from 42.32 to 36.91 one week after discharge and to 34.50 eight weeks after discharge in the intervention group. In the study by Duffy et al., however, the effects of time, group, and time-group interaction on anxiety variations were not significant one week and eight weeks after discharge (27). In the present study, the results of MANCOVA showed that the anxiety score at baseline influenced the anxiety variations throughout the studied time points. According to MANCOVA results, only the variation in the trait anxiety scores from one to eight weeks after discharge was not significant (*P* = .38). In this regard, the results of a study by BorimNejad et al., in Tehran, Iran on the effect of COPE program on the stress of mothers of premature infants showed that the mean stress scores decreased in the intervention group one week after discharge in comparison with the control group (P <0.01)(28). The results of the study by de Barros et al., in Brazil on the effect of group intervention on psychiatric features of patients with epilepsy showed that the quality of life in the intervention group improved (P = 0.003) and the implementation of the psychotherapy program reduced the anxiety level in the patients (P = 0.02), in addition, depression decreased in the intervention group (P <0.0001)(29).

In a study by Gürhopur et al., in Turkey, different results were obtained; therefore, the experimental group in comparison with the control group revealed significant improvements in knowledge (P <0.001), self-efficacy for seizure (P <0.001), and quality of life (P <0.001). The same significant improvements were also observed for parents’ awareness of epilepsy in the experimental group (P <0.001); however, the parents' anxiety in the experimental group also increased significantly (P <0.001)(30). Gürhopur believed that the parents’ increased level of anxiety was due to their enhanced knowledge about epilepsy during the program; a knowledge that made parents familiar with the threats of epilepsy. In the present study, on the contrary, the COPE program mostly focused on parents’ skills to adapt to their child’s disease.

Since the parents of children with neurological problems face many challenges on a daily basis and given that they sometimes have to manage their complex medical conditions, in addition to child rearing routines, how these parents respond to the needs and experiences of their child may facilitate their child’s adaptation to the chronic disease. This, in turn, comes true for the improved functioning of the whole family. Hence, research interventions should aim at facilitating the care transition from hospital to home.

**In conclusion**, the researcher claims that the presentation of the COPE program with no need for time spent by the nurse and with the use of simple facilities such as audio files along with training booklets and workbooks can reduce the anxiety level in mothers. Nurses taking care of such families should be able to recognize their educational and support needs, in addition to managing their treatment procedures. Nurses can support such families and assist them to learn strategies facilitating their children's adaptation. Further research is recommended to improve the quality of life among children with epilepsy. Future studies are also recommended to examine the effect of the COPE program on the anxiety and depression levels of other family members or on the behavior of children with epilepsy.
